# A review on the role of quinones in renal disorders

**DOI:** 10.1186/2193-1801-2-139

**Published:** 2013-04-01

**Authors:** Jennifer Madeo, Adeel Zubair, Frieri Marianne

**Affiliations:** Department of Medicine, Nassau University Medical Center, 2201 Hempstead Turnpike, East Meadow, NY 11554 USA

**Keywords:** Acute kidney injury (AKI), Chronic kidney disease, Co-enzyme Q, Nephrotoxic, Proton carriers, Reactive oxygen species (ROS)

## Abstract

Quinones are electron and proton carriers that play a primary role in the aerobic metabolism of virtually every cell in nature. Most physiological quinones are benzoquinones. They undergo highly regulated redox reactions in the mitochondria, Golgi apparatus, plasma membrane and endoplasmic reticulum. Important consequences of these electron transfer reactions are the production of and protection against reactive oxygen species (ROS). Quinones have been extensively studied for both their cytotoxic as well as cellular protective properties and they have been particularly useful in rational drug design. The role of quinones in medicine is explored in this literature review with a particular focus on renal diseases. Due to their high basal metabolism and detoxification role, the kidneys are particularly sensitive to oxidative stress. Regardless of the underlying etiology, ROS plays an important role in both acute kidney injury (AKI) and chronic kidney diseases (CKD). Depending on the oxidative state of the kidney, quinones can be nephrotoxoic or nephro-protective. Many factors play a role in the interaction between quinones and the kidney and the consequences of this are just beginning to be explored.

**Electronic supplementary material:**

The online version of this article (doi:10.1186/2193-1801-2-139) contains supplementary material, which is available to authorized users.

## Quinones in biology

**Figure 1:**
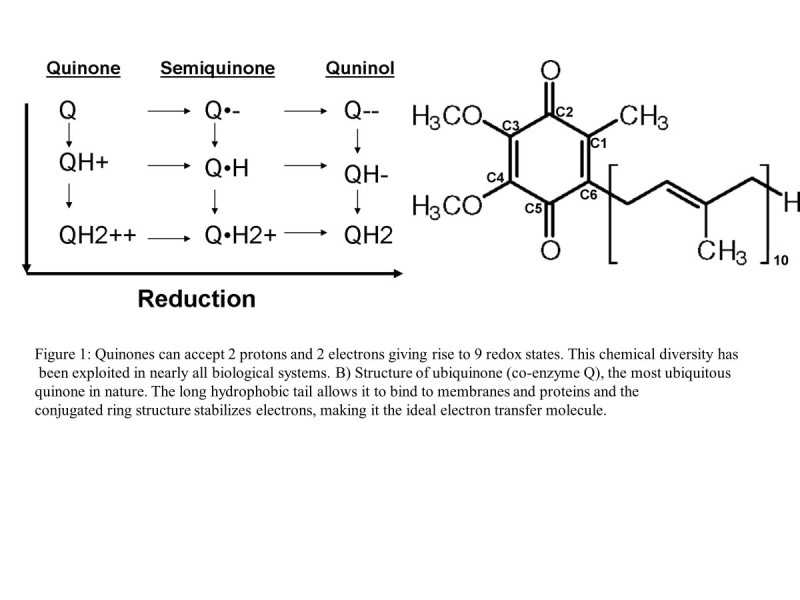
**Quinones can accept 2 protons and 2 electrons giving rise to 9 redox states.** This chemical diversity has been exploited in nearly all biological systems. B) Structure of ubiquinone (co-enzyme Q), the most ubiquitous quinone in nature. The long hydrophobic tail allows it to bind to membranes and proteins and the conjugated ring structure stabilizes electrons, making it the ideal electron transfer molecule.

Benzoquinones are a class of small molecules that contain two carbonyl groups on a benzene ring. They are ubiquitous in nature and constitute an important class of naturally occurring compounds found in plants, fungi and bacteria. As shown in Figure 1, benzoquinones have three oxygenation states, fully oxidized, free radical semi-quinone and fully reduced quinol. In addition, each of these states can accept up to two protons giving rise to a total of nine unique redox states (Rich & Bendall^1979^). Their unique stability, chemical diversity and conjugated structure make them ideal biological electron transfer mediators.

Ubiquinones (co-enzymes Q) are found in the cells of all aerobic organisms from the simplest bacteria to humans. They mediate important proton and electron transfer reactions in the mitochondria, chloroplasts, Golgi apparatus, endoplasmic reticulum and the plasma membrane of oxidative bacteria. Thus they are of key importance in producing energy during oxidative metabolism and photosynthesis. The electrons are ultimately used to reduce molecular oxygen to water releasing the energy necessary to sustain the life of the cell. Disruption of this process can lead to disease states. NADH:ubiquinone oxidoreductase (Complex 1), the largest multi-meric enzyme complex of the mitochondrial respiratory chain, is a well-studied example involved in many disease states including cancer (Albracht et al.^2011^). Dysfunction of this complex is the most common oxidative phosphorylation disorder in humans (Mimaki et al.^1817^). Leigh syndrome, a subacute necrotizing encephalomyelopathy is a well-known example of this (Leigh^1951^). Redox reactions mediated by benzoquinones are the source of potentially cytotoxic reactive oxygen species (ROS) including superoxide, hydrogen peroxide, and the hydroxyl radical. Quinone mediated ROS can cause cellular damage through alkylation reactions with lipids, proteins, and DNA (Bolton et al.^2000^). Depending on the particular system, quinones can act as anti-oxidants and protect healthy cells against ROS, or act as cytotoxic agents, generating ROS in unhealthy cancer cells. The generation of ROS and its cytotoxic effects depend on the inducing chemical, the host tissue, and other factors that likely influence the electrochemical environment. This has been observed with the effects of nanomaterials on cells and tissues. A review of the literature revealed that an inflammatory response and an increased production of ROS are common immune responses to nanomaterial use which has implications for drug delivery systems (Syed et al.^2012^). On the other hand, thin walled carbon nanotubes do not appear to produce cellular damage despite the fact that they produce ROS (Thurnherr et al.^2011^; Ogasawara et al.^2012^).

Quinones are of interest from a medical and toxicological prespective due to their unique reactivity and high prevalence in the environment (Ralph et al.^2011^; Monks & Jones^2002^). They undergo highly regulated redox reactions while bound to specific sites in integral membrane proteins such as the cytochrome b_6_f (Kurisu et al.^2003^), QFR (Q_P_,Q_D_) (Iverson et al.^2001^), succinate dehydrogenase (Yankovskaya et al.^2003^), DsbB (Zhou et al.^2008^) cytochrome bc1 oxidoreductase (Gao et al.^2003^) and photosynthetic reaction centers (Stowell et al.^1997^). One extensively studied system that has significantly increased our understanding the role of quinone electrochemistry in biology is bacterial reaction centers (Gunner et al.^2008^). Reaction centers (RCs) are large membrane-bound proteins that use the energy of a photon to catalyze a series of electron transfer reactions during the process of photosynthesis. Ubiquinone-10 simultaneously binds to 2 unique active sites and the protein mediates electron transfer between them (Stowell et al.^1997^; Zhu & Gunner^2005^). This inevitably produces protein-bound free radicals that must be properly sequestered to avoid cytotoxic damage (Madeo & Gunner^2005^). Studying this system has provided much insight how proteins have evolved to shift the energy of electron transfer reactions to fit the needs of the cell (Madeo et al.^2011^).

## Quinones in medicine

Quinones play an important role in medicine. Oxidative stress occurs when there is an imbalance between the production and quenching of free radicals from oxygen species. The mitochondria play a central role in the formation of excess ROS. Quinones can target the mitochondria and re-establish electron transfer in deficiency states. For example, in antiphospholipid syndrome, treatment with co-enzyme Q has been shown been shown to alter mitochondrial dynamics resulting in lower oxidative stress and slowing of the accelerated atherosclerosis (Perez-Sanchez et al.^2012^). Co-enzyme Q has also been shown to prevent retinal cell apoptosis when given as eye drops in mouse models of kainate-induced retinal damage (Lulli et al.^2012^). Quinones are also being investigated for the treatment of mitochondrial diseases (Enns et al.^2012^) as well as age-related diseases (Skulachev et al.^2009^). In one small open-label study, patients with terminal genetic mitochondrial diseases were treated with a para-benzoquinone (alpha-trocotrienol quinone) for 13 weeks as an emergency treatment protocol. This therapy modified disease progression in 90% of the patients by increasing quality of life and improving central nervous system oxidation state (Enns et al.^2012^). This quinone is currently undergoing clinical trials for diseases such as Friedreich’s ataxia, Leigh Syndrome, and Tourette syndrome.

Depending on their redox state, quinones can act as both pro and anti-oxidants and their action in the mitochondrial is highly dependent on their concentration. Ubiquinone-10, a fat soluble quinone, is found in particularly high concentrations in the mitochondria of cells in the brain, heart, liver, and kidney. Deficiencies in ubiquinone-10 have been associated with the pathology of a diverse number of diseases, including encephalopathy, myopathy, male infertility, and nephrotic syndrome (Villalba et al.^2010^; Quinzii & Hirano^2010^; Balercia et al.^2009^). Accelerated arteriosclerosis is driven by the proinflammatory response and ubiquinones in the plasma appear to play a protective role against coronary artery disease. Co-enzyme Q, by interfering with the production of ROS, protects circulating lipoproteins including LDL from oxidation thereby slowing the progress of coronary artery disease (Morre & Morre^2011^; Tsai et al.^2011^). During arteriosclerosis, oxidized LDL binds to an endothelial cell receptor triggering a signal transduction pathway that activates NADPH oxidase resulting in the generation of ROS. The oxidative insult to the endothelium plays a key role in the pathogenesis of arteriosclerosis. This pathway was interrupted in endothelial cells pretreated with co-enzyme Q diminishing endothelial oxidative damage (Tsai et al.^2011^). Any systemic inflammatory disease has the potential to cause oxidative damage due to the increase in aerobic metabolism and thus could benefit from these protective effects. An important example is in systemic lupus erythematosus where accelerated atherosclerosis plays an important role (Kurien & Scofield^2008^; Frieri^2012^).

NAD(P)H:quinone oxidoreductase 1 (NQO1) is an enzyme important in maintaining oxygen homeostasis. It is a flavoprotein that catalyzes the reduction of quinones, vitamin E, and nitrogen oxides. These reduction reactions generate natural antioxidants (such as reduced form of vitamin E and ubiquinol) and are important for both intrinsic and exogenous chemical detoxification (Ross et al.^2000^). Such reactions can protect against exogenous quinones that would otherwise promote the generation of ROS. On the other hand, depending on the specific initial quinone, these reduction reactions can generate labile products that react with molecular oxygen to generate ROS and cause DNA and protein alkylation that can thus destroy the cell. This is the principle behind NQO1 anti-tumor drugs. Compounds of the quinone family such as mitomycin C are efficiently bioactivated by NQO1, a cytosolic two-electron reductase. This enzyme activates anti-tumor pro-drugs and is thus a necessary agent for their anti-tumor activity (Begleiter et al.^1997^). Due to the fact that NQO1 is expressed in particularly high levels in tumor cells, these drugs target cancerous cells before becoming toxic to normal cells. Drugs that target succinate: quinone reductase/Complex II, another enzyme found in particularly high levels in tumor cells, is also being developed based on this principle (Ralph et al.^2006^).

Dysregulated ROS production can be cytotoxic, particularly to cancer cells that have a high metabolism. Cancer cells are particularly sensitive to insults in redox homeostasis. ROS production can activate genes involved in detoxification and regulated cell death (Dhakshinamoorthy et al.^2000^). Many cancer cells maintain a moderate level of oxidative stress and current investigations are aiming at exploiting this for targeted cell death (McCarty et al.^2010^). Quinones, due to their redox cycling ability, are being used to exploit this process and thus represent a large class of antitumor drugs currently under investigation (Siegel et al.^2012^; Sagar et al.^2010^). These drugs particularly target cancer cells rather than healthy cells. The mechanism of death appears to involve ATP depletion, calcium imbalance, and protein degradation (Verrax et al.^2011^). The reduction of quinones by NQO1 plays an important role. There is still a lot of research that needs to be done to understand the role anti-oxidants play in signal transduction and altering gene expression. The better we can understand these mechanisms the better we can design specific drugs that target specific cancers. For example, NQO1 triggers a NF-ĸB mediated signal transduction pathway that regulates apoptosis; NF-ĸB is an important transcription factor in the pathogenesis of many cancers, including breast cancer (Zubair & Frieri^2012^). One study showed that expression of NQO1 and NF-ĸB were inversely related in breast tissue implicating a unique pathway that can be targeted for rational drug design (Jamshidi et al.^2012^).

Due to their anti-oxidant effects, quinones are also currently being investigated as part of the treatment of head trauma (Kalayci et al.^2011^) and neurological diseases like Parkinson disease (Kerr^2010^; Rakoczi et al.^2009^), Huntington disease, (Hyson et al.^2010^) and Alzheimer disease (Yang et al.^2010^). On the other hand quinones are involved in the induction of cancer and neurodegenerative disease (Zahid et al.^2010^; Cavalieri & Rogan^2006^). Quinone derivatives of estrogens, benzene, and dopamine have been showed to promote formation of DNA adducts that initiate mutations leading to cancer as well as neurodegenerative diseases (Zahid et al.^2010^). Plastoquinol, an anti-oxidant found in chloroplasts, is being investigated for its involvement in the suppression of age-related pathologies such as declining immunity, balding, cataracts and osteoporosis (Skulachev et al.^2011^). Aging is associated oxidative stress, thus quinones have a potential role in modulating the process (Soares et al.^2011^). Lastly, quinones are still being investigated for their antimalarial effect (Qu et al.^2010^; Jacquerioz & Croft^2009^).

## Pathogenesis of renal disease

Hypoxia and cellular reactions to oxidative stress are at the heart of the complex pathogenesis of both acute and chronic kidney diseases (Nangaku^2006^; Aksu et al.^2011^). Acute kidney injury (AKI) affects millions of people and has been associated with increased mortality and hospital length of stay as well as puts the patient at risk for future chronic kidney disease (CKD) (Singbartl & Kellum^2012^). Regardless of the etiology, the pathology of AKI is intimately tied to renal microcirculation and mitochondrial dysfunction resulting in disruption of oxygen homeostasis, uncontrolled inflammation, and production of ROS (Kunzendorf et al.^2010^). ROS can promote cell death by both necrosis and apoptosis. ROS production is triggered by ischemic/reperfusion injury (Eltzschig & Eckle^2011^), antibiotics such as aminoglycosides (Ali et al.^2011^), contrast induced renal injury (Oudemans-van Straaten^2005^), rhabdomyolysis (Boutaud & Roberts^2011^) and toxic materials (Gobe & Crane^2010^; Chen et al.^2011^). Therapeutic interventions aimed at inhibiting these pathological redox reactions is an active area of current research. NAD(P)H:quinone oxidoreductase is a redox enzyme found in particularly high levels in renal tubular cells and likely contributes to pathogenesis of renal failure. Renal specific oxidases are known to be involved in ROS medicated damage in kidney mesangial cells (Orient et al.^2007^). Natural anti-oxidant agents have shown some promise in ameliorating aminoglycoside toxicity (Ali et al.^2011^; Sayed-Ahmed & Nagi^2007^) and cisplatin-induced nephropathy (Mukhopadhyay et al.^2012^). The role of antioxidants in the management of AKI is an ongoing area of research (Koyner et al.^2008^).

Oxidative stress is known to play an important role in the development of renal diseases such as glomerulonephritis, drug-induced nephrotoxicity, and CKD (Okamura & Himmelfarb^2009^). CKD is associated with mitochondrial dysfunction that leads to an imbalance between ROS and the natural anti-oxidants that normally quench these pathological free radicals. In particular hypertensive nephropathy and proteinuria have been directly linked to salt-induced oxidative stress (Nagase et al.^2007^; Varagic et al.^2010^). One study showed that high salt intake activates the renin-angiotensin system, urinary protein excretion and nitroxidative markers in rat models (Varagic et al.^2010^). By using a novel beta-blocker with anti-oxidant function, this study showed that the kidney damage associated with the oxidative stress is independent of the hemodynamic damage of hypertension induced by the high salt diet. Salt loading increases activity of plasma membrane NADPH oxidase in the renal parenchyma, an enzyme important for production of ROS (Kitiyakara et al.^2003^). Oxidation of LDL by mesangial cells is important in the pathogenesis of lipoprotein glomerulopathy which can lead to rapid progressive renal failure (Sakatsume et al.^2001^). ROS production also appears to play a key role in diabetic nephropathy and could be induced by long-standing hyperglycemia (Forbes et al.^2008^). Angiotensin II facilitates ROS production in kidney mesangial cells which helps to explain the renoprotective role of ACE-inhibitors and angiotensin receptor blockers in these disorders (Gorin et al.^2004^). Another interesting role of anti-oxidants in renal disease is aristolochic acid 1 (AA1) nephropathy. This disease is associated with tubulointerstitial fibrosis and urothelial carcinoma following ingestion of AA1 (De Broe^2012^). Renal reduction, which is important for AA1 clearance, generates a toxic metabolite. One study showed that pre-treatment of mouse tubular epithelium with a NQO1 inhibitor increased the oxidized metabolite of AA1 in renal tissue leading to attenuation of AA1 induced nephropathy (Chen et al.^2011^). This highlights the importance of this enzyme in regulating renal oxidation and homeostasis and illustrates how genetic polymorphisms can contribute to a person’s susceptibility to this disease.

## Quinones in renal disease

Quinones can be toxic or protective to the kidneys. The difference depends on the products produced by reduction and the oxidative state of the kidney. Stable fully oxidized quinones are less reactive and their production can quench other cellular toxins such as ROS. On the other hand, partially reduced semiquinones are free radicals that can damage DNA and lead to cell death (see Figure 1). Thymoquinone (TQ), a compound derived from Nigella sativa, shows strong anti-oxidant properties against gentamicin (GM)-induced nephrotoxicity. In a study using a rat model with GM-induced nephrotoxicity, TQ supplementation lowered BUN, creatinine and total nitrate/nitrite. It also increased glutathione peroxidase activity and ATP to match control values. TQ supplementation also prevented GM-induced renal histopathology. Based on these findings, the mechanism of TQ prevention is likely related to its ability to decrease oxidative stress by preserving the activity of the anti-oxidant enzymes (Sayed-Ahmed & Nagi^2007^). With evidence for a similar anti-oxidant mechanism, TQ also protects the kidney from vancomycin and cisplatin (Ragheb et al.^2009^; Basarslan et al.^2012^). Due to its anti-inflammatory, anti-allergic, and anti-carcinogenic effects, TQ has been used in traditional medicine for a diversity of diseases such as cancer, diabetes, and asthma (Khan et al.^2011^; Sankaranarayanan & Pari^2011^). One study observed that TQ reduced sepsis related morbidity and mortality in mice challenged with endotoxin Gram-negative bacteria. TQ reduced mortality by 80-90%, improved both kidney and liver function and it also lowered inflammatory biomarkers (Alkharfy et al.^2011^).

Hemodialysis (HD) and peritoneal dialysis (PD) are associated with increased oxidative stress which may be related to low levels of co-enzyme Q in the blood. The major risk of this oxidative stress is accelerated cardiovascular disease, the leading cause of death in dialysis patients. One observational study, comparing dialysis patients to healthy controls, found an association between lower co-enzyme blood levels and higher oxidative stress in dialysis patients (Mehmetoglu et al.^2012^). This has not been a consistent finding and larger, higher powered studies are needed before this relationship can be accepted (Gokbel et al.^2011^; Lippa et al.^1994^). Another small study followed 36 HD patients for 6 months and showed that co-enzyme Q10 administration was partially effective for suppressing the oxidative stress in HD patients (Sakata et al.^2008^). A recent Cohrane review examined the benefits and harms of giving antioxidants to CKD, dialysis, and kidney transplant patients (Jun et al.^2012^). Although the analysis did not detect a significant decrease in cardiovascular and all-cause death, there was evidence that antioxidant therapy significantly reduced development of end-stage of kidney disease in CKD patients and lowered serum creatinine levels. Significant adverse effects were not observed. The findings of these observational studies have led to the currently ongoing clinical trial assessing the effect of co-enzyme Q-10 on oxidative stress and systemic inflammation in HD patients and have encouraged larger, appropriately powered studies. Another renal protective role for quinones has been observed with cold-storage injury, which occurs with deceased donor kidneys that are stored at cold temperatures prior to transplantation. Oxidative damage, from mitochondrial superoxide generation, plays a role in the tissue injury that occurs during storage and re-warming (Mitchell et al.^2010^). Mitoquinone is a ubiquinone analog with a conjugated hydrophobic anion that targets the mitochondria and has been shown to protect against cold storage damage of renal tubular cells by preventing mitochondrial dysfunction (Mitchell et al.^2011^).

The toxicity of quinones to the kidney appears to depend on a variety of factors including genetic polymorphisms and the disease state of the individual. NQO1 is found, in particular high levels in podocytes indicating that these cells play a pivotal role in the oxidative state of the kidney (Tamura et al.^2012^; Marshall et al.^2006^). The likely role is a detoxifying enzyme, protecting the kidney from chemically reactive metabolites filtered through the glomerulus. Mitomycin C is an anticancer agent that is particularly toxic to the kidney and known to cause hemolytic uremic syndrome (Zappa et al.^2003^). It is a quinone that requires cellular reduction to activate its cytotoxic effects. Due to its ability to reduce quinones, NQO1 in podocytes could play a major role in the pathogenesis of renal toxicity and mitomycin C-induced hemolytic uremic syndrome. Injury to the glomerular filtration mechanism is the primary damage, leading to a cascade of deleterious events including microangiopathic hemolytic anemia and thrombocytopenia (Zappa et al.^2003^). The high expression of NQO1 explains, in theory, a selective toxicity towards podocytes. Another mechanism may be the formation of covalent protein quinol-thioether adducts. Glutathione (GSH) conjugates of hydroquinone (HQ) and 2-bromohydroquinone (2-BrHQ) produce severe renal proximal tubular necrosis in rats (Kleiner et al.^1998^).

Podocytes are the major cell of the glomerular filter. They are highly differentiated and quiescent cells involved in the pathology of numerous kidney diseases (Durvasula & Shankland^2006^; Garg & Holzman^2012^). The glomerular filtrate must traverse capillary endothelium, basement membrane, and the podocyte layer before reaching Bowman’s space. Podocytes also produce vascular endothelial growth factor (VEGF), which regulates glomerular endothelial permeability, thus playing an important role in the kidney interactions with potential toxins (Kretzler et al.^1998^). Expression of VEGF has been shown to be important in many renal diseases, particularly lupus nephritis (Frieri et al.^2012^). An acquired primary dysregulation of podocytes is described in collapsing idiopathic focal segmental glomerulosclerosis and in HIV-associated nephropathy (Barisoni et al.^1999^), another disease driven by unregulated oxidative stress.

Primary coenzyme Q10 deficiency is considered to be the only treatable mitochondrial disorder, since some patients have a response to oral coenzyme Q-10 (Salviati et al.^2005^). The disease usually manifests with nephropathy and encephalomyopathy (Montini et al.^2008^). It is caused by mutations in the enzymes of the coenzyme Q10 synthetic pathway. It has been linked with focal segmental glomerulosclerosis due to dysfunctional mitochondria in the podocytes. One study using a translational mouse model showed that giving probucol (a lipid soluble anti-oxidant) prevented an otherwise lethal glomerulopathy (Falk et al.^2011^). This drug resulted in higher levels of co-enzyme Q in kidney and liver compared to supplementation alone. The benefit appears to be due to effects on signaling pathways that alter gene expression resulting in reduction of oxidative stress. Since not all patients with co-enzyme Q deficiency respond to supplementation, alternative therapies targeting the signaling pathways that mediate oxidative stress are needed.

Another study described the effects of long-term coenzyme Q10 supplementation in a patient with coenzyme Q10 deficiency (Diomedi-Camassei et al.^2007^). Progressive recovery of renal function and resolution of nephritic syndrome were observed during the 50 months of treatment. Corticosteroids and immunosuppressive drugs were not needed. On the other hand, a second patient was treated with co-enzyme Q after the development of CKD and no change in renal function was observed. Thus, early administration of coenzyme Q10 appears to be important for the resolution of renal symptoms.

## Conclusions

This literature review highlights the relationship between benzoquinones, oxidative stress, and renal disease. Quinones are small electron transfer molecules, found in virtually all cells that undergo aerobic metabolism, that play an important role in oxidative stress. Quinones have a diverse role in medicine, including anti-cancer agents and anti-aging and arteriosclerosis. Quinones can be renal toxic or renal protective. Thymoquinone protects the kidney from toxic effects of drugs, including gentamicin, vancomycin and cisplantin. Mitoquinone can protect against cold storage damage of deceased donor kidneys. Mitomycin C is an anticancer agent that is particularly toxic to renal podocytes because it targets the detoxifying enzyme NQO1. Primary coenzyme Q10 deficiency also results in kidney damage primarily due to dysfunctional mitochondria in the podocytes. CoQ10 administration was partially effective for suppressing the oxidative stress in dialysis patients. Although the role of co-enzyme Q in CKD and ESRD is not fully established, encouraging observational studies and lack of significant adverse effects encourage future investigations. Many factors play a role in the interaction between quinones, oxidative stress and kidney physiology. Future studies, including clinical trials, can help to better unravel this role and improve the management of patients with or at risk for kidney diseases.

## Authors’ original submitted files for images

Below are the links to the authors’ original submitted files for images. Authors’ original file for figure 1
