# Supplementary figures and images for: A review on the role of quinones in renal disorders
Source: Springerplus. 2013 Apr 1;2(1):139. doi: 10.1186/2193-1801-2-139 (PMC3618882; doi:10.1186/2193-1801-2-139)

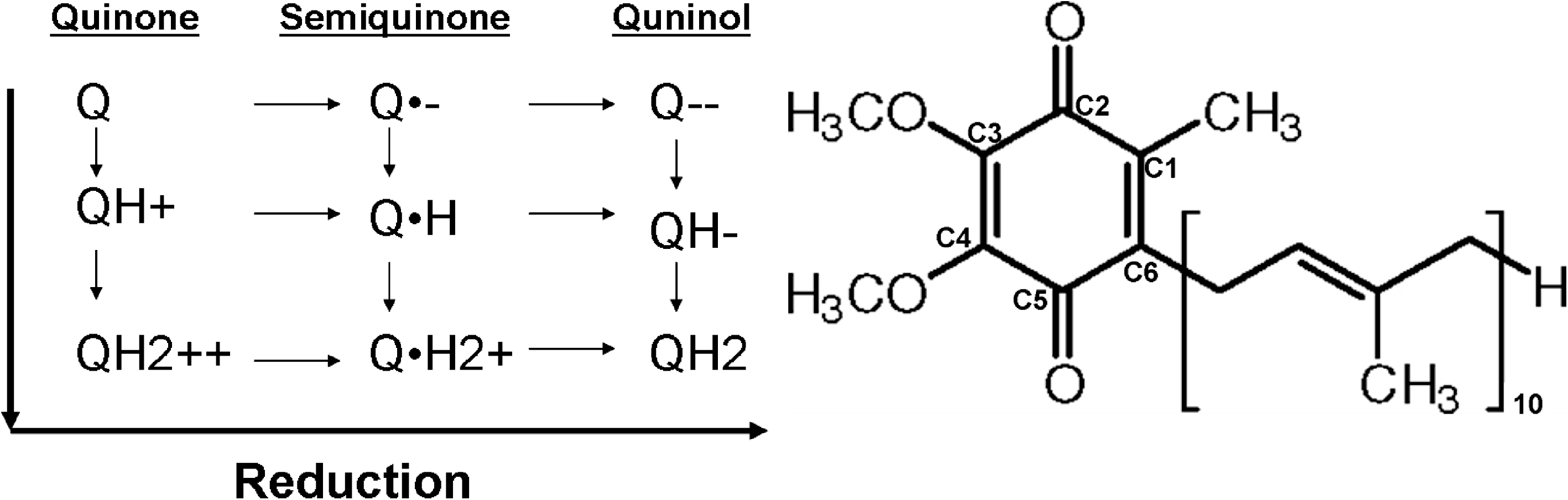

Supplement: Supplementary file 1 — Authors’ original file for figure 1 [file 40064_2012_194_MOESM1_ESM.tiff]
